# The role of VND transcription factors in xylem vessel development and secondary wall formation

**DOI:** 10.1111/nph.70327

**Published:** 2025-06-26

**Authors:** Ulrike Lehmann, René Schneider

**Affiliations:** ^1^ Plant Physiology Department, Institute of Biochemistry and Biology University of Potsdam 14476 Potsdam‐Golm Germany; ^2^ Collaborative Research Center 1644 University of Potsdam 14476 Potsdam‐Golm Germany

**Keywords:** metaxylem, microtubule rearrangements, programmed cell death, protoxylem, ROP GTPases, secondary cell walls, vascular‐related NAC domain transcription factors, xylem vessel development

## Abstract

Transitioning from aquatic to terrestrial life required specialized vascular tissues, with xylem enabling water transport and structural support. Vascular‐related NAC (NAM, ATAF1/2, CUC2) domain (VND) transcription factors regulate xylem development by orchestrating secondary cell wall biosynthesis and programmed cell death. This review examines recent advances in understanding VND function in xylem vessel development, focusing on their structure, regulatory networks, downstream targets, and molecular events in *Arabidopsis thaliana*, while highlighting key open questions in vessel differentiation.

**Table:** 

	Contents	
	Summary	2034
I.	Introduction	2034
II.	Vascular‐related NAC domain transcription factors	2035
III.	Direct transcriptional targets of VNDs	2035
IV.	Spatio‐temporal expression of VNDs	2038
V.	Inducible VND systems	2038
VI.	VND7‐induced sequence of cellular events	2038
VII.	Outlook	2039
	Acknowledgements	2039
	References	2040

## I. Introduction

As plants moved from aquatic to terrestrial habitats, they evolved vascular xylem tissue to cope with dry, unpredictable conditions by enabling water transport from roots to shoots. Xylem consists of four main cell types: (1) tracheary elements (TEs) for lateral and longitudinal water conduction, (2) vessel elements for efficient end‐to‐end flow, (3) mechanically supportive fibers, and (4) living parenchyma cells for storage and metabolism. Acting as a combined tissue, xylem significantly reinforces structural integrity, maintains turgor pressure, and enables stomatal function, allowing up to 95% of transported water to be lost in exchange for CO₂. Developing primary xylem consists of two vessel types: protoxylem (PX), with spiral or banded secondary cell walls (SCWs), and metaxylem (MX), featuring thicker walls with regularly spaced pits. These patterns arise from templated SCW deposition, governed by spatio‐temporal regulation of microtubules (MTs), the plasma membrane (PM), and the trafficking machinery (Xu *et al*., 2022). Despite structural differences across species and xylem cell types, their development follows a conserved program after fate is set: elongation halts, SCWs form, and programmed cell death (PCD) is initiated. This is driven by irreversible transcriptional changes and remodeling of the cytosol, the cytoskeleton, and the cell wall, ending with vacuolar disassembly to enable water transport (Xu *et al*., 2022).

Although key transcription factors (TFs) and their downstream targets involved in xylem and SCW formation have been identified, significant gaps remain – especially in understanding the dynamics and transcriptional regulation underlying endogenous xylem development. In contrast to experimentally induced systems, the sequence of molecular and cellular events driving this transition under physiological conditions is still poorly resolved. This mini‐review summarizes recent insights into the transcriptional switch preparing xylem vessels for water conduction, with a focus on vascular‐related NAC domain (VND) TFs. Given the concise format, we exclude xylem fiber development, regulated by other NAC TFs (NST1‐3/SND1), and refer to dedicated reviews (Schuetz *et al*., 2013; Růžička *et al*., 2015; Sun *et al*., 2022; Xu *et al*., 2022). We also outline key open questions and possible experimental approaches.

## II. Vascular‐related NAC domain transcription factors

NAC (NAM, ATAF1/2, and CUC2) domain TFs form a large family in plants. They are characterized by highly conserved N‐terminal DNA‐binding and dimerization domains and variable C‐termini that mediate transcriptional regulation. These proteins play pivotal roles in development and stress responses, including xylem differentiation and PCD (Nakashima *et al*., 2012; Singh *et al*., 2021; Dong *et al*., 2024).

The role of VND TFs in xylem vessel development was first identified using *Zinnia elegans* cell cultures, which show xylem vessel differentiation upon hormonal induction. Gene expression analyses during this transition identified Z567, a TF homologous to *Arabidopsis thaliana* (Arabidopsis) NAC proteins, later classified within the VND subfamily (Demura *et al*., 2002). Similar studies in Arabidopsis identified seven VNDs (VND1–VND7), all upregulated upon hormone‐induced xylem vessel formation (Kubo *et al*., 2005; Yamaguchi *et al*., 2008). These findings underscore the functional specialization of VNDs in regulating vessel differentiation.

**Fig. 1:**
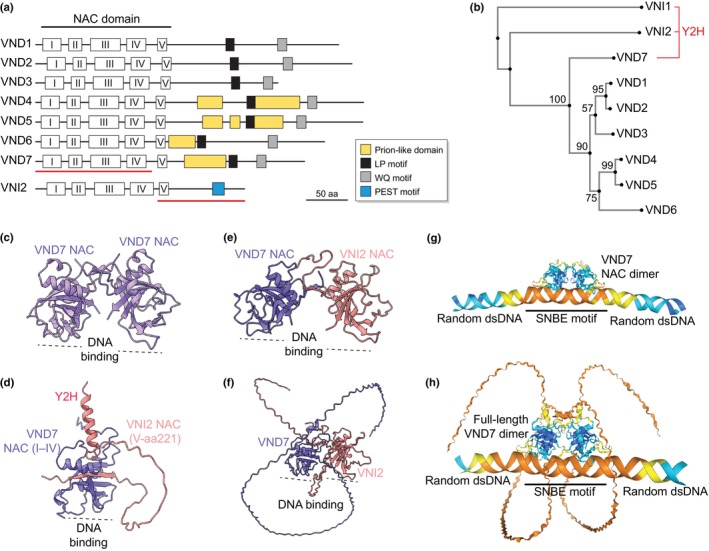
Vascular‐related NAC domain (VND) structure and phylogeny in *Arabidopsis thaliana*. (a) Domain structure of VNDs and VNI2, highlighting N‐terminal NAC subdomains (I–V) and prion‐like domains (yellow) predicted by the PLAAC webtool (Lancaster *et al*., 2014). (b) Phylogenetic tree of VNDs and VNIs, constructed using the Mafft webtool (https://mafft.cbrc.jp/alignment/server/). (c–f) Dimerization models: (c) VND7 NAC domain homodimer, (d) truncated VND7 (NAC subdomains I–IV) interacting with truncated VNI2 (subdomain V to C‐terminus, red lines in A) from yeast two‐hybrid (Y2H) studies, (e) VND7‐VNI2 NAC domain dimer, and (f) full‐length VND7‐VNI2 complex. DNA‐binding sites face downward (dashed lines). (g, h) DNA‐binding models of VND7 NAC dimers (g) and full‐length VND7 dimers (h) interacting with a 19‐bp secondary wall NAC binding element (SNBE) motif flanked by random DNA. Models were generated using AlphaFold3 and visualized in ChimeraX.

The N‐terminal NAC domain of VNDs consists of five subdomains (I–V), facilitating nuclear localization (III, IV), DNA binding (IV, V), and dimerization (Fig. 1a). Dimerization preferences vary: VND7 NAC domains readily dimerize with NAC domains of VND1–VND3, whereas VND6 NAC domains primarily form homodimers (Yamaguchi *et al*., 2008). Whether full‐length VNDs utilize these possibilities *in planta* remains unexplored but could reveal combinatorial VND functions during vessel development.

Sequence alignment of VNDs, together with NAC TFs VNI1 and VNI2 identified via yeast two‐hybrid (Y2H) screening (Yamaguchi *et al*., 2010b), revealed two distinct clades: VND1–VND3 and VND4–VND7, with VND6 and VND7 as more divergent members (Fig. 1b). This classification reflects variations in the C‐termini, which modulate transcriptional activity. In VND7, transcriptional activity depends on conserved LP and WQ boxes – named after the amino acids leucine (L), proline (P), tryptophan (W), and glutamine (Q) that define these motifs – as demonstrated by reduced ectopic SCW deposition in Arabidopsis plants expressing C‐terminally truncated VND7 compared to the full‐length protein (Ko *et al*., 2007; Yamaguchi *et al*., 2008, 2010b).

Prion‐like domains in the C‐termini of VND4–7 underlie their phylogenetic distinction and may promote liquid–liquid phase separation (LLPS; Wu & Li, 2024), a process increasingly recognized in plants (Jung *et al*., 2020; Zhu *et al*., 2024). While their precise function remains unclear, LLPS can decouple gene regulation from enzymatic activity and confer responsiveness to physical cues, such as temperature, osmotic stress, and pH (Jung *et al*., 2020; Liu *et al*., 2023). These features may indicate a potential role for prion‐like domains in modulating VND4–VND7 function during vessel developmental or in response to environmental changes.

Since its introduction, AlphaFold has become a key tool in structural biology (Abramson *et al*., 2024). Using this approach, all VND NAC domains are predicted to form dimers (Fig. 1c–f), with each monomer composed of a twisted antiparallel β‐sheet flanked by N‐ and C‐terminal helices. The VND7 interactor VNI2 was initially identified through a coincidental interaction between truncated VND7 (NAC subdomain I–IV) and VNI2 (subdomain V to C‐terminus), allowing VNI2's subdomain V to complement the missing region in VND7 (Fig. 1d). While this artificial configuration is unlikely *in vivo*, full‐length VND7–VNI2 interactions have since been confirmed in multiple assays, supporting their physiological relevance (Yamaguchi *et al*., 2008; Ailizati *et al*., 2021).

AlphaFold predicts stable dimers for both the NAC domains and full‐length forms of VND7 and VNI2 (Fig. 1e,f). Transient transfection assays in Arabidopsis rosette leaves and protoplasts revealed that VND7 and VNI2 form a complex that mutually inhibits transcriptional activity, mediated by VND7's C‐terminus and VNI2's N‐terminus (Yamaguchi *et al*., 2010b; Ailizati *et al*., 2021). AlphaFold modeling tentatively suggests that this inhibition may not result from disrupted DNA binding, as VND7–VNI2 dimers appear to retain configurations similar to VND homodimers (Fig. 1c,f). Instead, inhibition could potentially involve protein degradation or other speculative mechanisms associated with their largely unfolded C‐termini (Fig. 1f). Further studies will be necessary to identify additional interactors, determine their stoichiometry in complexes, and clarify the molecular mechanisms underlying this proposed regulatory switch.

## III. Direct transcriptional targets of VNDs

**Fig. 2:**
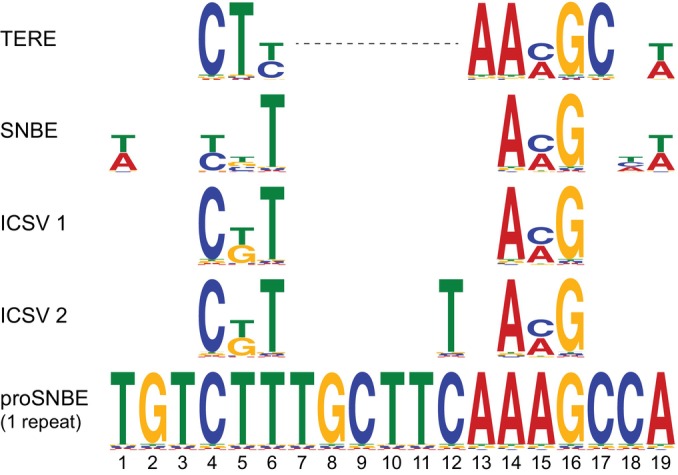
VND7 target sequences in *Arabidopsis thaliana*: TE‐regulating *cis*‐element (TERE), secondary wall NAC binding element (SNBE), and Ideal Core Structure for VND7 (ICSV). Motif comparison between the 11‐bp TERE, the 19‐bp SNBE, two versions of the 13‐bp ICSV1 and ICSV2, and one 19‐bp repeat of the synthetic triple SNBE promoter. Motif comparison generated using the Tomtom tool (Gupta *et al*., 2007). TE, tracheary element. VND, vascular‐related NAC domain.

Many VND7 target promoters contain the TE‐regulating *cis*‐element (TERE), associated with vessel differentiation (Pyo *et al*., 2007). VND7, VND6, and the fiber‐specific NAC TF SND1 bind the secondary wall NAC binding element (SNBE) to regulate SCW formation (Zhong *et al*., 2010). Fluorescence correlation spectroscopy and systematic evolution of ligands by exponential enrichment identified an optimal VND7‐binding motif, termed the Ideal Core Structure for VND7 (ICSV; Tamura *et al*., 2019). TERE, SNBE, and ICSV share core sequence features and are considered variants of the same *cis*‐element (Fig. 2), as illustrated by the artificial SNBE promoter derived from the *Xylem Cysteine Protease* (*XCP1*), which includes this motif in triplicate and can serve as a model *cis*‐element for PX‐specific regulation (McCarthy *et al*., 2011; De Meester *et al*., 2018).

AlphaFold predicts that VND7 NAC and full‐length dimers specifically bind the TERE/SNBE/ICSV motif within otherwise random DNA, underscoring its structural uniqueness (Fig. 1g,h). These motifs are found in promoters of VND7‐induced genes, such as *CESA4*, *CESA8*, *MYB46*, *MYB83*, *COBL4*, and *KNAT7* (Zhong *et al*., 2010; Yamaguchi *et al*., 2010a; Yamaguchi *et al*., 2011; Table 1). TERE is rare but specific, while SNBE and ICSV are more permissive and present also in many non‐xylem promoters, for example in *pWUS*, *pWOX5*, and *pUBQ10*, raising questions about how xylem‐specific regulation is achieved. Other *cis*‐elements likely contribute, as indicated by the VND7‐mediated repression of VNI2 (Ailizati *et al*., 2021). Single‐cell regulatory studies may help clarify these mechanisms.

**Table 1:** Selected promoters of *Arabidopsis thaliana* containing TE‐regulating *cis*‐element (TERE), secondary wall NAC binding element (SNBE), and Ideal Core Structure for VND7 (ICSV) motifs.

VND7 binding motifs:								
SNBE (TA)NN(CT)(TCG)TNNNNNNNA(AC)GN(ACT)(AT)								
ICSV C(GT)TNNNNNNNA(AC)G or C(GT)TNNNNNTNA(AC)G								
TERE CT(TC)NAA(AC)GCN(AT)								
Promoter	Gene locus	Function	SNBE	No. of hits in 2 kb before ATG	ICSV	No. of hits in 2 kb before ATG	TERE	No. of hits in 2 kb before ATG
*pSNBE*	–	Artificial promoter consisting of three SNBE elements	Yes	3	Yes	3	Yes	3
*pXCP1*	AT4G35350	Associated with SCW formation	Yes	7	Yes	1	No	0
*pXCP2*	AT1G20850		Yes	4	Yes	3	Yes	1
*pCESA4*	AT5G44030		Yes	1	No	0	Yes	1
*(pCESA7)*	AT5G17420		Yes	5	Yes	2	No	0
*pCESA8*	AT4G18780		Yes	5	Yes	1	No	0
*pMYB46*	AT5G12870		Yes	13	Yes	5	No	0
*pMYB83*	AT3G08500		Yes	9	Yes	5	No	0
*pMYB103*	AT1G63910		Yes	5	Yes	1	No	0
*pLBD15*	AT2G40470		Yes	7	Yes	3	Yes	1
*pLBD30*	AT4G00220		Yes	6	Yes	2	Yes	1
*pKNAT7*	AT1G62990		Yes	7	Yes	1	No	0
*(pVND6)*	AT5G62380	Regulators of xylem vessels	No	0	Yes	1	No	0
*pVND7*	AT1G71930		Yes	4	Yes	2	No	0
*(pVNI1)*	AT5G09330	Interactors/Inhibitors of VND7	Yes	5	Yes	2	No	0
*pVNI2*	AT5G13180		Yes	5	No	0	No	0
*(pCESA1)*	AT4G32410	Primary wall‐associated Cellulose synthases	Yes	7	Yes	2	No	0
*(pCESA3)*	AT5G05170		Yes	6	Yes	1	No	0
*(pCESA6)*	AT5G64740		Yes	5	Yes	1	No	0
*(pWUS)*	AT2G17950	Stem cell maintenance (shoot)	Yes	9	No	0	No	0
*(pWOX5)*	AT3G11260	Stem cell maintenance (root)	Yes	3	No	0	No	0
*(pUBQ10)*	AT4G05320	Housekeeping	Yes	1	Yes	2	No	0

Analysis of motif abundance within 2‐kb upstream of the ATG start codon. Motif occurrences were identified using the ‘find similar DNA sequences’ function of SnapGene®. Underlined promoters have been shown to be directly targeted by VND7, whereas those in parentheses lack experimental confirmation. The UBQ10 promoter serves as references. SCW, secondary cell wall; TE, tracheary elements.

Comprehensive analyses of the TERE/SNBE/ICSV motifs in Arabidopsis promoters have identified a suite of likely direct VND7 targets (Pyo *et al*., 2007; Zhong *et al*., 2007, 2008, 2010; Soyano *et al*., 2008; Ko *et al*., 2009; McCarthy *et al*., 2009; Yamaguchi *et al*., 2011; Endo *et al*., 2015; Taylor‐Teeples *et al*., 2015; Turco *et al*., 2019):

1. Transcriptional regulation: *MYB46*, *MYB83*, and *MYB103*, *LBD15* and *LBD30*, *KNAT7*, and other MYB‐domain, zinc finger, AP2, and bHLH TFs.
2. Cytoskeleton and vesicle transport: Several genes code for kinesin‐like proteins, such as FRA1, and MT‐interacting proteins, such as MIDD1/RIP4 and MAP65‐8.
3. Cell wall biosynthesis and modification: Cellulose synthase genes *IRX5/CESA4*, *IRX1/CESA8*, xylan synthesis subunits *IRX10*, *IRX14*, lignin‐related cinnamate 4‐hydroxylase (*C4H*), several peroxidases, laccase genes *LAC4/IRX12* and *LAC17*, and other factors, such as *IRX13/FLA1*, glycosyltransferase genes, and members of the glycosyl hydrolase, peroxidase, and UDP‐xylose synthase gene families.
4. Programmed cell death: *XCP1* and *XCP2*, caspase gene family members, such as metacaspase (*MS)9*, as well as several other peptidase genes.

A major gap in the VND7 transcriptional network is the limited identification of effectors involved in cytosolic and cytoskeletal remodeling. Notably, *ROP7*, *ROP11*, and *ROPGAP3* have been identified as direct VND7 targets (Zhong *et al*., 2010; Yamaguchi *et al*., 2011). *ROP11*, essential for MX pit formation, provides a promising link to SCW patterning in general and is now increasingly studied through computational and biophysical approaches (Oda & Fukuda, 2012; Jacobs *et al*., 2019, 2020, 2022; Deinum & Jacobs, 2024).

## IV. Spatio‐temporal expression of VNDs

**Fig. 3:**
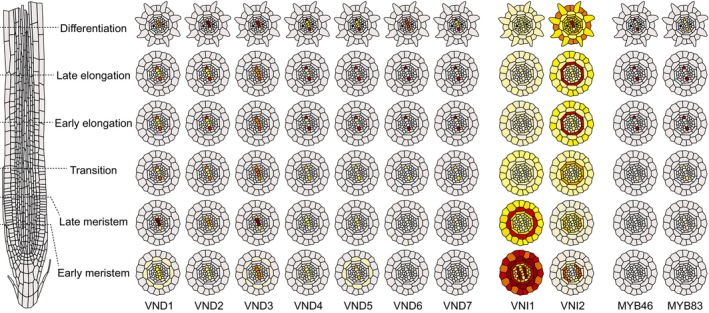
Spatio‐temporal expression profiles of vascular‐related NAC domains (VNDs) and xylem‐specific transcription factors (TFs) in *Arabidopsis thaliana*. Based on scRNA‐seq data from seven datasets, mapped onto an idealized root layout (Root Cell Atlas, https://rootcellatlas.org/).

The phylogenetic grouping of VNDs (Fig. 1b) is reflected in their distinct C‐termini and supported by their spatial–temporal expression in Arabidopsis roots (Fig. 3). Transcriptional reporters based on yellow fluorescent protein fused to a nuclear localization signal (YFP‐NLS) reveal that VND1–VND3 are active in early procambial cells of the root meristem (Kubo *et al*., 2005), consistent with their established roles in cotyledon xylem differentiation (Tan *et al*., 2018) and MX differentiation under water stress and abscisic acid treatment (Ramachandran *et al*., 2021). However, their relative contributions and dimerization status remain unclear.

*VND4–VND7* are expressed from the transition zone upward in the PX, while *VND6* also appears in MX vessels. *VNI2*, a translational inhibitor of *VND7*, follows a pattern consistent with its putative antagonistic role, being strongly expressed where PX does not form. Although based mainly on Arabidopsis protoplast data and lacking *in planta* evidence, VNI2 may be downregulated upon PX fate commitment, allowing VND4–VND7 to activate MYB46 and MYB83 (Ailizati *et al*., 2021). Although *VND7* regulation is better understood, the roles of other *VNDs* in vessel differentiation require further *in planta* studies. scRNA‐seq holds promise (von der Mark *et al*., 2024) but is limited by protoplastation, excluding late PX and MX stages. This may obscure the roles of other VNDs and unknown TFs in xylem maturation, although scRNA‐seq remains key for mapping early gene regulatory networks.

## V. Inducible VND systems

Inducible overexpression systems – such as 35S‐driven, dexamethasone‐, and estradiol‐inducible constructs – have been essential for identifying other downstream VND6/7 targets (Kubo *et al*., 2005; Yamaguchi *et al*., 2010a; Coego *et al*., 2014; Turco *et al*., 2019; Xu *et al*., 2022). The inducible VND7 system, in particular, allows dynamic imaging of SCW patterning and transdifferentiation, providing an alternative to deep‐tissue xylem vessel imaging (Watanabe *et al*., 2015, 2018; Schneider *et al*., 2017, 2021; Vukašinović *et al*., 2017; Wightman, 2023; Higa *et al*., 2024). This system has revealed many PX‐related genes, including *CSI1*, EXOCYST components, *KTN1*, MT nucleation factors, and ubiquitination regulators (Li *et al*., 2016; Schneider *et al*., 2017, 2021; Vukašinović *et al*., 2017; Phookaew *et al*., 2024). These findings support scRNA‐seq data and enhance computational modeling efforts (von der Mark *et al*., 2024).

While inducible VND6/7‐driven systems have identified key effectors, they provide limited insight into alternative regulatory pathways. All VNDs and other NAC TFs can induce SCW deposition when overexpressed (Mitsuda *et al*., 2007; Endo *et al*., 2015), yet single VND knockouts show no major vessel defects (Kubo *et al*., 2005), suggesting redundancy. CRES‐T suppression of VND6 or VND7 reduces MX or PX formation, respectively, while constitutive VND1–VND5 expression delays growth, highlighting their shared roles (Endo *et al*., 2015). All VNDs can induce *VND7* in transactivation assays (Endo *et al*., 2015), pointing to a regulatory cascade with VND7 as a central node. Their sequence divergence and distinct expression profiles (Fig. 3) indicate a need to explore their functional interactions in endogenous xylem contexts.

## VI. VND7‐induced sequence of cellular events

**Fig. 4:**
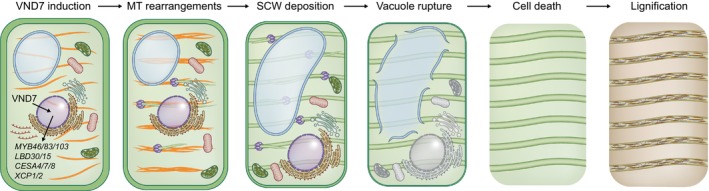
Order of events induced by VND7. Overview of protoxylem (PX) transdifferentiation following fate commitment, including VND7 induction, upregulation of direct targets, microtubule (MT) rearrangements (transverse and equally spaced bundles), secondary cell wall (SCW) deposition (mostly cellulose), vacuole swelling and rupture, nuclear breakdown, cytoplasmic clearance, cell death, and lignification, culminating in fully functional water‐conducting cells.

Inducible VND7 overexpression uncovers a sequence of events driving PX‐like SCW formation (Fig. 4), involving *CESA4/7/8* and regulated cortical MT rearrangements, increasingly investigated by *in silico* modeling (Watanabe *et al*., 2015, 2018; Schneider *et al*., 2017, 2021; Jacobs *et al*., 2020, 2022, 2025). *CESA4* and *CESA8* are direct transcriptional targets of VND7, while *CESA7* – although likely an indirect VND7 target – is involved in triggering a rapid and irreversible PX fate switch (Turco *et al*., 2019). MT rearrangements begin *c*. 10 h after VND7 induction (Schneider *et al*., 2017, 2021), but the cause of this delay in SCW formation remains unclear.

Plant‐specific Rho‐related GTPases of plants (ROPs) are thought to coordinate cytoskeletal dynamics with processes at the plasma membrane (Deinum & Jacobs, 2024). In VND6‐controlled MX, active ROP11 marks pit sites via ROPGAP/ROPGEF interactions that were successfully modeled by reaction–diffusion frameworks (Oda & Fukuda, 2012; Nagashima *et al*., 2018; Jacobs *et al*., 2019, 2020; Deinum & Jacobs, 2024). ROP11 recruits MIDD1 and KINESIN‐13A to locally disassemble MTs, forming SCW‐free pit zones (Oda *et al*., 2010; Oda & Fukuda, 2013; Higa *et al*., 2024). Disrupting this system blocks pit but not PX band formation, suggesting that ROP11 supports but does not initiate PX patterning (Oda & Fukuda, 2012; Higa *et al*., 2024). *VND7* directly regulates *ROP11* and *ROP7*, although up to eight *ROPs* may shape xylem development through, for example, interactions with dynamic MTs (Brembu *et al*., 2005; Li *et al*., 2016). A recent study shows that KNAT7‐mediated control of the actin polymerizer FORMIN11 enables the switch between MX pits – where FORMIN11 is repressed – and PX band patterns, where its derepression leads to F‐actin accumulation and the formation of wide gaps partitioning SCW bands (Kijima *et al*., 2025).

After VND7 induction, PCW CESAs (CESA1/3/6‐like) are replaced by SCW CESAs (Watanabe *et al*., 2018), although the detailed mechanisms remain unclear. Lignification occurs after PCD and relies on both internal and neighboring cell inputs (Pesquet *et al*., 2013; Schuetz *et al*., 2014; De Meester *et al*., 2018). LAC4 detection *c*. 18 h post‐VND7 induction suggests that it happens late, but unclear PCD timing blurs its distinction from SCW deposition. PCD is key for vessel maturation, with vacuole‐stored hydrolytic enzymes (XCP1, XCP2, and MC9) clearing the cytoplasm after tonoplast rupture (Funk *et al*., 2002; Avci *et al*., 2008). While expressed early, their activation timing and the triggers for vacuolar rupture remain unclear. The inducible VND7 system will help dissect these events by synchronizing development for targeted analysis.

## VII. Outlook

Future research should look beyond VND7 and the VND family to uncover the roles of other TFs in vessel differentiation – especially in secondary xylem, which makes up most plant biomass and holds greater agro‐economic importance. While VND function is better understood in primary xylem, studies in poplar suggest similar mechanisms in secondary xylem (Ohtani *et al*., 2011). Key goals include clarifying TF interactions, complex stoichiometry, and their roles in SCW patterning and PCD. Advanced tools like CRISPR‐Cas, single‐cell transcriptomics, and protein interaction studies (Zheng *et al*., 2025), along with computational modeling and live‐cell imaging, will help link gene regulation to the physical shaping of vessels and may inform strategies to enhance crop vascular systems.

## Competing interests

None declared.

## Author contributions

UL and RS jointly contributed to drafting and editing the manuscript, preparing figures and tables, conducting literature research and evaluation, and formulating future research directions. Both authors reviewed and approved the final version of the manuscript.

## Disclaimer

The New Phytologist Foundation remains neutral with regard to jurisdictional claims in maps and in any institutional affiliations.
